# Industry and Bioethics: What Price the Relationship?

**DOI:** 10.1371/journal.pmed.0030281

**Published:** 2006-06-27

**Authors:** Mark Boyd, Wendy Rogers

**Affiliations:** **1**The Flinders University of South AustraliaBedford ParkAustralia

We read with interest the article by Mackie et al. entitled “Lessons on Ethical Decision Making from the Bioscience Industry” [ 1]. The authors recognise some study limitations, including the possibility of social desirability bias, but fail to address other limitations that in our view seriously weaken the paper.

Firstly, there is no discussion regarding an understanding of the use of the term “ethics” by the authors and bioscience companies. There appears to be an assumption that “ethics” is a straightforward term whose meaning would be agreed by all those engaged in the field. However, the requirements of business ethics, for example, may differ significantly from the requirements of healthcare ethics. When one considers issues such as priority-setting, environmental concerns, sales and marketing, and the like, it is not clear that the ethical imperatives of the bioscience and healthcare industries substantially overlap. This study engages with what might be defined as procedural issues, but ignores substantive philosophical issues. The latter may have been beyond the scope of the paper, but if this were the case, it should have been acknowledged.

Secondly, there is no comment upon the authors' industry links. These are disclosed in their listing of their competing interests and include receipt of industry funding, direct links with companies subject to study, and funding awarded by some of the involved companies after the study. However, there is no discussion of the potential for these links to interfere with study conduct and interpretation. The authors do acknowledge the debate regarding bioethics and links with industry, but such acknowledgements cannot realistically compensate for the conflict of interest faced in the conduct of this particular study. Despite the growing literature on these links, there is no comprehensive analysis of industry-associated bioethics research [ 2, 3]. We cannot therefore confidently claim that there is an observable industry bias in such research. There is, however, overwhelming evidence that bias favourable toward funders occurs in medical research and healthcare prescribing [ 4–6]. It would therefore seem naive to believe that bioethicists are in some way immune from factors that demonstrably lead to bias in other disciplines.

In addition to these omissions, the accompanying Perspectives commentary [ 7] neglects to discuss the implications of the conflicts of interest for the design, conduct, conclusions, and interpretation of the study. There was an allusion to these conflicts, but in this context we would have expected a review to be far more explicit regarding the potentially crucial importance of such conflicts.

Ethicists are wooed by industry precisely because their views and opinions carry weight. This currency will soon become valueless unless researchers, authors, reviewers, and journal editors take a strong stand for intellectual honesty and self-critique in the presence of conflicts of interest.
